# On the Sequelæ and Prophylaxis of Simple Tonsillar Hypertrophy in Children

**Published:** 1876-01

**Authors:** Beverly Robinson

**Affiliations:** Surgeon to Manhattan Eye and Ear Hospital (Department of the Throat)


On the Sequelæ and Prophylaxis of Simple Tonsillar Hypertrophy in Children.—*By Dr. Beverly Robinson, Surgeon to Manhattan Eye and Ear Hospital (Department of the Throat).*—What are the tonsils?

Histologists tell us they resemble in structure other lymphatic organs of the body. At the same time they inform us that they are characterized dy differential features, which approach them to glandular structures. On the one hand, we find in the walls surrounding the central cavity of each one of the masses, which conglomerated form the entire organ, a number of closed follicles, similar to those that form a component part of Peyer's patches in the small intestine; on the other, we have a number of tortuous tubes, not unlike, in form and structure, the ducts of acinous glands, and these tubes open either directly upon the mucous surface of the tonsil, or else into the lacuna of the lobules. Now, from this structure proceed two distinct functions: 1, the formative function, which relates to the constitution of the blood;

2, the glandular, which determines the secretion of a clear, viscid fluid. This fluid, when poured into the buccal cavity, by the contraction of the surrounding muscles in the effort of deglutition, serves to imbibe and lubricate the alimentary bolus.

The tonsils are situated, as you are aware, between the pillars of the fauces on either side, and when of normal size, although easily seen if the mouth be opened and the throat inspected, are not prominent enough to materially diminish the diameters of the isthmus, or interfere with essential vital functions.

How changed this statement becomes whenever the tonsils are diseased. First, and considered as lymphatic organs, their influence upon the transformation of the white blood corpuscles is imperfect or null. Secondly, the secretion from the glandular structure is altered. It rapidly becomes cloudy and fetid, is apt to flow less easily from the orifices of the follicles, and in many instances these latter become blocked up completely with a white sebaceous substance of cheesy consistence, which is also soon rendered putrescent. As a consequence the alimentary bolus, though lubrified in its passage through the bucco-pharyngeal opening, carries with it a quantity of morbid secretion, which rapidly sets up dyspeptic trouble, and absorbed into the circulation leads finally to unhealthy nutrition. Owing to the position of the tonsils, we can at once perceive how, in cases of chronic enlargement, no matter what may be their intimate or structural characters, several important functions are morbidly affected in a greater or less degree. If the tonsils enlarge towards the median line, the faucial opening is often greatly diminished. At times, and when the hypertrophy is exaggerated in this direction, deglutition is fatiguing and occasionally painful. If the enlargement also takes place in the upward direction, partial obstruction of the posterior nares and compression of the Eustachian orifices are natural sequences, and hence we have difficult respiration and imperfect hearing, and on account of the relative immorbidity of the soft palate, the voice takes that nasal and muffled intonation so familiar to every medical practitioner. * * *

A well-known physiological law is expressed in the following terms: whenever an organ of the body, during its period of growth, is thrown into disuse, or cut off completely from its normal function by some mechanical obstacle, deformation and arrest of development, followed by atrophy, are natural sequelæ. Let us make the application of this law. Owing to the passage of a less quantity of air through the nasal passages, in the condition under consideration, a diminished volume also enters the larynx and lungs.

What is the result? The nasal passages do not increase in capacity proportionately with the growth of the child, and this lack of growth affects in an equal degree the palatine arch; consequently we find the olfactive and gustatory senses restricted. Again, the lungs do not receive a sufficient supply of oxygen, a certain number of their terminal vesicles are not expanded at all during ordinary inspirations, and those vesicles that are filled are insufficiently so, and the chemical blood changes which take place in the pulmonary structure are but very imperfectly accomplished. Besides, atmospheric pressure in the interior of the lungs, owing to the rarefaction of the air, does not fully counterbalance that from the exterior, and sinking in, with a peculiar deformation of the chest walls, is produced. This visible alteration in the chest is sufficiently notable to be in the estimation of some authors as pathognomonic of tonsillar enlargement, as a somewhat analogous appearance is of rickets. The physical development and proper growth of children thus affected is manifestly seriously impeded. Their nutrition languishes and their muscular energy and activity are markedly diminished.

Further, we may add, by compression of the large vessels of the neck cerebral circulation is at times incompletely performed, the brain becomes dull and apathetic, less inclined to exert itself, and suffers concomitantly with the body, from a condition which is local in origin and may at any time, with rare exceptions, be remedied by a ready and safe operatory procedure.

In the prophylactic treatment of chronic enlargement of the tonsils in children, we attach great importance to first, the habit of cold bathing.

We all know how readily children "catch cold," and how frequently this catching cold, as it is termed, manifests itself upon the mucous membrane lining the faucial opening. It is to frequently repeated inflammations of the throat, more than to any other single cause, that is due the disease under consideration.

To guard our little patients efficiently, then, against the recurrence of colds, let us endeavor by bathing in water of low temperature to activate the functions of the skin, and thus to lessen the likelihood of localized mucous congestion.

Mothers, nurses, and even some of our professional brethren, are too much disposed to underrate the value, or be fearful of cold ablutions. They recognize how useful a preventive agent against colds they have ever close at hand whenever adults are in question, but they do not believe this to be true of children; or if they acknowledge that cold baths might prove useful, they dread putting their knowledge into practice. They seem to consider that a small child is not sufficiently strong, that its vital forces are not energetic enough to be able to resist such a heroic method of treatment. They tremble for the safety of their nurslings, or children of a more advanced age, and cannot bear to carry out this rational therapeutic system.

And yet is it not manifest in our ever-varying climate, where extremes of temperature so closely approximate, that in spite of every precaution being exercised which is dictated by maternal solicitude, children must of necessity be more or less frequently exposed to all the evils that result from draughts, or from cold, or excessive humidity in the atmosphere? Our object, then, should be to protect them efficiently from the adverse effects of our trying climate. Now, *hot*, or even *tepid bathing*, is, we believe, one of the main causes of recurring congestion or inflammation of the throat. And, we may ask, can it be otherwise? Take a child of a relatively feeble and lymphatic constitution, and subject it to bad hygienic influences, viz: surround it with an insufficient or vitiated supply of air, give it improper food, or cover it with badly-adapted clothing, and will you not find that it gradually becomes more markedly strumous and sickly? Warm bathing is to be ranked in the same category. It is enervating, and takes away from bodily vigor.

The skin, it is true, is actively congested during the period of the bath, and its capillary circulation greatly augmented, but just so soon as the ambient cold air infringes again upon the cutaneous surface, either directly or through the habitual wearing apparel, the blood supply is driven with increased force (owing to the rapid contraction of the small vessels of the integument) towards the internal viscera and mucous linings, which, in their turn, become congested, and remain so more or less *constantly*, unless by a superabundance of clothing the body is kept in an unnatural state of heat.

If the temperature of the water used in bathing is as low or lower than that of the surrounding atmospheric medium, what a different physiological action takes place.

A temporary shock follows immersion or the use of the sponge filled with water, after which there is a short period when the surface temperature of the body is lowered, and then a natural warmth or glow takes place, the skin is reddened, its capillary circulation is heightened, and not merely in a temporary manner, but shortly becomes so permanently, and the interior organs are relieved of an overload of blood and greatly activated in their several functions. In the event of the natural warmth, which almost invariably succeeds the use of cold water for bathing purposes (unless it be continued too great a length of time on each occasion), not manifesting itself, we ought to have recourse to *friction*. And rubbing gently at first, and soon with a firmer pressure, the entire trunk and limbs, will greatly stimulate the action of the skin. The rubbing should always proceed from the extremities towards the heart, or in the direction of the venous blood-flow. The use of some fatty, or oleaginous substance of bland, unirritating nature, is an excellent adjunct in carrying out the above treatment, and is especially of service where the skin is dry, offers a slightly scaly or furfuraceous aspect, and so gives evidence of imperfect nutrition, or lack of healthy power. Of course, in the above indications of prophylaxis, we would naturally include careful attention to the periodical, but not too frequent, use of *pure* soap. The selection of this article has its importance, and we shall therefore particularly recommend Pears' transparent glycerine soap, which is manufactured in London, and is one of the best soaps of which we have cognizance.

Another subject to which we claim attention is care of the feet. They should be kept warm and dry. Cold, moist feet are an habitual and most efficient cause in producing hypertrophy of tonsils. Nowadays our people have finally been awakened to the necessity of warm and appropriate clothing for small children, if they hold to their continuance in good health. Happily, therefore, naked arms, legs, shoulders and chests are not so frequently encountered as formerly. * * *

Colds may also be rendered somewhat less frequent in young children, and attacks of acute tonsillitis may, we are told, be occasionally aborted by putting a plug of cotton wool in either ear whenever they go into the open air during a period of excessive cold or high winds. The habit is recommended in a late number of the *London Practitioner* (and the physiological explanation given), and lauded far above the custom of surrounding the neck with mufflers of any description.

One other point is worthy of consideration, viz: Cold is taken just as easily by the rapid passage from a very cold, or damp atmosphere, to one where the temperature is excessively dry or elevated, as by the sudden change from the latter conditions to those first mentioned. We should be careful therefore to counsel nurses—

1st. Not to keep the nursery at a too high temperature, and always to have a basin, pitcher, or bucket full of fresh, pure water, with the surface exposed to the air contained in the room.

2d. Not to allow children to approach a fire-place or register immediately upon entering or leaving the house.

3d. Not to keep extra wraps on their little charges, when at home, a moment longer than is absolutely essential.

Practical attention to the preceding rules, and also to what we have written with respect to cold bathing, care of the feet, etc., as prophylactic measures of hypertonsillar growth, will be found, we are firmly convinced, of unquestionable utility.

Artificial Dilitation of the Cervix in the Obstinate Vomiting of Pregnancy.—In the proceedings of the Obstetrical Society of Boston (*Boston Medical and Surg. Journal*) Dr. Sinclair referred to cases recently reported by Dr. Copeman. Dr. Copeman was called to a patient six months advanced in pregnancy. In endeavoring to break the membranes, he failed after two or three efforts. He retired for an hour to consult, and in the mean time the vomiting had ceased. The patient did well without further interference. In another case, a pregnancy of two months, abortion was proposed, and the cervix was dilated with the same result. In another patient, eight months along, a like effect resulted from the same treatment. Graily Hewitt, in commenting upon the above cases, says that vomiting due to version or flexion of the uterus is removed by dilatation. He says he has accomplished the same thing by elevating the womb. Dr. Sinclair had had a case in Charlestown, of a woman who had been vomiting from the sixth week to the fourth month. She was put in the knee and elbow position with good effect.

Dr. Minot reported a case in which a sponge tent stopped the symptoms completely, much to the indignation of the woman, who had hoped to abort. In another case, seen with Dr. Clarke, Dr. Minot had applied tincture of iodine. Dr. Putnam, in consultation with Dr. Clarke, proposed removing the contents of the

